# Comparative analysis of neuroinvasion by Japanese encephalitis virulent and vaccine viral strains in an *in vitro* model of human blood-brain barrier

**DOI:** 10.1371/journal.pone.0252595

**Published:** 2021-06-04

**Authors:** Cécile Khou, Marco Aurelio Díaz-Salinas, Anaelle da Costa, Christophe Préhaud, Patricia Jeannin, Philippe V. Afonso, Marco Vignuzzi, Monique Lafon, Nathalie Pardigon

**Affiliations:** 1 Unité de Recherche et d’Expertise Environnement et Risques Infectieux, Groupe Arbovirus, Institut Pasteur, Paris, France; 2 Unité de Neuro-Immunologie Virale, Institut Pasteur, Paris, France; 3 Unité d’Epidémiologie et Physiopathologie des Virus Oncogènes, Institut Pasteur, CNRS UMR 3569, Paris, France; 4 Unité des Populations Virales et Pathogenèse, Institut Pasteur, Paris, France; Lewis Katz School of Medicine, Temple University, UNITED STATES

## Abstract

Japanese encephalitis virus (JEV) is the major cause of viral encephalitis in South East Asia. It has been suggested that, as a consequence of the inflammatory process during JEV infection, there is disruption of the blood-brain barrier (BBB) tight junctions that in turn allows the virus access to the central nervous system (CNS). However, what happens at early times of JEV contact with the BBB is poorly understood. In the present work, we evaluated the ability of both a virulent and a vaccine strain of JEV (JEV RP9 and SA14-14-2, respectively) to cross an *in vitro* human BBB model. Using this system, we demonstrated that both JEV RP9 and SA14-14-2 are able to cross the BBB without disrupting it at early times post viral addition. Furthermore, we find that almost 10 times more RP9 infectious particles than SA14-14 cross the model BBB, indicating this BBB model discriminates between the virulent RP9 and the vaccine SA14-14-2 strains of JEV. Beyond contributing to the understanding of early events in JEV neuroinvasion, we demonstrate this *in vitro* BBB model can be used as a system to study the viral determinants of JEV neuroinvasiveness and the molecular mechanisms by which this flavivirus crosses the BBB during early times of neuroinvasion.

## Introduction

*Flaviviruses* such as Japanese encephalitis virus (JEV) are arthropod-borne viruses (arbovirus) that are transmitted through the bite of an infected mosquito and may cause serious human diseases [1]. JEV is the main causative agent of viral encephalitis in South East Asia, with an annual incidence of around 68 000 cases [2]. About 30% of those are fatal, and half of the survivors present neurological sequelae [3]. Although no specific treatment against JEV exists [3], vaccines have been developed: the live-attenuated JEV SA14-14-2 strain, obtained empirically after several passages of the JEV SA14 virulent strain in primary hamster kidney cells [4], as well as a recombinant and an inactivated vaccines, both based on the JEV SA14-14-2 strain [5, 6]. Although highly efficient, rare cases of post-vaccine encephalitis have been reported [7], suggesting that the vaccine strain JEV SA14-14-2 can, in isolated cases, be neurovirulent in humans.

JEV has a positive-sense RNA genome encoding a single polyprotein flanked by two untranslated regions (UTR) at the 5’ and 3’ ends. This polyprotein is co- and post-translationally cleaved into three structural proteins (capsid C, membrane prM and envelope E) involved in viral particle assembly and antigenicity and seven non-structural proteins (NS1, NS2A, NS2B, NS3, NS4A, NS4B and NS5) involved in genome replication, viral particle assembly and evasion of innate immunity [1]. Due to an error-prone NS5 polymerase that frequently introduces mutations in the viral genome during replication, a *Flavivirus* population is not clonal, but rather a mix of multiple viral genomic species (aka quasispecies) [8, 9].

JEV is a neuroinvasive and neurovirulent virus. It is associated with neuroinflammation of the central nervous system (CNS) [10], and disruption of the blood-brain barrier (BBB), as shown *in vivo* in murine and simian models. Expression levels of tight junction proteins involved in maintaining BBB functions such as occludin, claudin-5 and zonula occludens 1 (ZO-1) are significantly decreased in symptomatic JEV-infected mice, suggesting physical disruption of the BBB [11]. It seems, however, that BBB disruption occurs after infection of the CNS cells in a mouse model of JEV-induced encephalitis [11] and that inflammatory response of infected astrocytes and pericytes plays a key role in BBB leakage [11–13], which taken together suggest that JEV can cross the BBB before disrupting it. Indeed early studies of JEV-infected mouse brains demonstrated that the virus was transported across the cerebral endothelium by endocytosis [14]. Vesicular transport of cellular cargoes through endothelial cells is known as transcytosis [15], but it is unclear whether this mechanism also applies to the transport of JEV.

In contrast to virulent JEV strains such as RP9, the vaccine strain SA14-14-2 was shown to be essentially non-neuroinvasive and non-neurovirulent in weanling ICR mice, but is still highly neurovirulent in neonates. The JEV SA14-14-2 genome contains 57 nucleotide differences positioned along the genome when compared to the parental strain SA14, leading to 25 amino-acid substitutions [16]. Mutations in the E and M proteins seem to attenuate JEV neurovirulence [17–19], while mutations in the 5’ UTR, capsid C and NS1-NS2A protein coding regions have been found to attenuate JEV neuroinvasiveness in a mouse model [18, 20, 21]. Despite the identification of these attenuating mutations, the specific amino-acids contributing to the attenuation of JEV SA14-14-2 are unknown.

Although encephalitis incidents have occurred after vaccination with the SA14-14-2 JEV strain, no virus could be recovered from them [7]. Whether these neurological adverse events originated from virus reversion to a virulent phenotype, a specific viral neuroinvasive and neurovirulent sub-population or from host determinants is also unknown [7]. In any case, the JEV vaccine strain, although much less neurovirulent and neuroinvasive than its parental counterpart, must have crossed the BBB in order to reach the CNS and initiate encephalitis.

The BBB is the physical and physiological barrier between the brain and the blood compartments in vertebrates, and it is comprised of a network of different cell types including the brain microvascular endothelium along with pericytes, astrocytes, microglia and the basement membrane [22]. Several BBB models have been developed in order to facilitate studies on the biology and pathophysiology of its diverse components, as well as to evaluate drug transport to the brain [23]. The brain microvascular endothelial cell line hCMEC/D3 exhibits a stable growth and endothelial marker characteristics that makes it suitable to form a reproducible and easy-to-grow BBB *in vitro*. hCMEC/D3 monolayers displays good restricted permeability to paracellular tracers and retains most of the transporters and receptors present on *in vivo* BBB [24]. Accordingly, hCMEC/D3 cells have been used to investigate host-pathogen interactions with human pathogens that affect the CNS [25, 26].

In the present study, we used an *in vitro* human BBB model consisting of hCMEC/D3 human endothelial cells cultivated on permeable supports above SK-N-SH human neuroblastoma cells to evaluate and compare the ability of both a virulent and a vaccine strain of JEV (JEV RP9 and SA14-14-2, respectively) to cross this BBB model.

## Material and methods

### Cell lines and JEV strains

Human endothelial cells hCMEC/D3 [24], were maintained at 37°C on rat collagen diluted at 100μg/mL in water (Cultrex; catalog no. 3443-100-01) in EndoGro medium (Merck Millipore; catalog no. SCME004) supplemented with 5% heat-inactivated fetal bovine serum (FBS) and 10mM HEPES buffer (Sigma-Aldrich; catalog no. 83264). hCMEC/D3 cells can form tight junctions when cultured for 6 days at 37°C. Human neuroblastoma cells SK-N-SH (ATCC HTB-11) were maintained at 37°C in Dulbecco modified Eagle medium (DMEM) supplemented with 10% FBS. *Cercopithecus aethiops* monkey kidney Vero cells were maintained at 37°C in DMEM supplemented with 5% FBS. *Aedes albopictus* mosquito cells C6/36 were maintained at 28°C in Leibovitz medium (L15) supplemented with 10% FBS.

A molecular cDNA clone of JEV genotype 3 strain RP9 was kindly provided by Dr. Yi-Ling Lin [27]. This plasmid was modified in our laboratory as previously described [28], generating pBR322(CMV)-JEV-RP9, and used by transfecting C6/36 cells with Lipofectamine 2000 (Life Technologies; catalog no. 11668–019) to produce infectious virus. Once a cytopathic effect was visible, viral supernatant was collected and used to infect C6/36 cells. Because we found hCMEC/D3 monolayers very sensitive to any change of medium, we ensured that viruses were produced from cells grown in the same medium as the one used to grow endothelial cells (EndoGro medium). CD.JEVAX^®^ (JEV SA14-14-2) vaccine was kindly provided by Dr. Philippe Dussart (Institut Pasteur of Phnom Penh, Cambodia), and reconstituted with 500μL of DMEM. Two hundred and fifty μL of reconstituted vaccine were used to infect Vero cells for 7 days. Viral supernatants were collected and used to infect C6/36 cells cultivated in EndoGro medium supplemented with 2% FBS. Both JEV RP9 and SA14-14-2 viral supernatant stocks were collected 3 days after infection and the infectious titer was determined in Vero cells by focus-forming assay (see below).

### Antibodies

Mouse hybridomas producing the monoclonal antibody 4G2 anti-*Flavivirus* E protein were purchased from the ATCC (catalog no. HB-112), and a highly-purified antibody preparation was produced by RD Biotech (Besançon, France). Mouse monoclonal anti-JEV NS5 antibody was kindly provided by Dr. Yoshiharu Matsura [29]. Horseradish peroxidase (HRP)-conjugated goat anti-mouse IgG antibody was obtained from Bio-Rad Laboratories (catalog no. 170–6516). Alexa Fluor 488-conjugated goat anti-mouse IgG antibody was obtained from Jackson ImmunoResearch (catalog no. 115-545-003).

### Evaluation of JEV neuroinvasive capacity

hCMEC/D3 cells (5.10^4^/well) were seeded on 12-well Transwell^®^ permeable inserts (Corning; catalog no. 3460) in EndoGro medium supplemented with 5% FBS and placed at 37°C for 5 days. SK-N-SH cells (2.10^5^/well) were seeded in 12-well tissue culture plates in EndoGro supplemented with 2% FBS. Permeable inserts containing hCMEC/D3 cells were then transferred in these culture plates and medium was replaced by EndoGro medium supplemented with 2% FBS. Aliquots of virus were diluted the next day in 50μL of EndoGro medium supplemented with 2% FBS, heated at 37°C and then added to the cells. Cells were incubated at 37°C until collection.

### Focus-forming assay (FFA)

Vero cells were seeded in 24-well plates (10^5^/well). Ten-fold dilutions of virus samples were prepared in DMEM and 200μL of each dilution was added to the cells. The plates were incubated for 1h at 37°C. Unabsorbed virus was removed and 800μL of DMEM supplemented with 0.8% carboxymethyl cellulose (CMC), 5 mM HEPES buffer, 36 mM sodium bicarbonate, and 2% FBS were added to each well, followed by incubation at 37°C for 48h for JEV RP9 or for 72h for JEV SA14-14-2. The CMC overlay was aspirated, and the cells were washed with PBS and fixed with 4% paraformaldehyde for 20 min, followed by permeabilization with 0.1% Triton X-100 for 5 min. After permeabilization, the cells were washed with PBS and incubated for 1h at room temperature with anti-E antibody (4G2), followed by incubation with HRP-conjugated anti-mouse IgG antibody. The assays were developed with the Vector VIP peroxidase substrate kit (Vector Laboratories; catalog no. SK-4600) according to the manufacturer’s instructions. The foci were then counted in each well manually. The viral titers were expressed in focus-forming units (FFU) per milliliter.

### Lucifer Yellow (LY) permeability assays

LY dye migration through the BBB monolayers was performed as previously described [25, 26]. Briefly, Transwell^®^ inserts containing hCMEC/D3 monolayers were transferred in culture wells containing 1.5 mL of Hanks’ Buffered Salt Solution (HBSS) supplemented with 10 mM of HEPES buffer, 1 mM of sodium pyruvate and 50μM of LY (Sigma-Aldrich; catalog no. L0144). The culture medium inside the Transwell^®^ inserts was replaced with 500μL of HBSS buffer containing 50μM of LY. Cells were incubated at 37°C for 10 min. Permeable inserts were then transferred in culture well containing 1.5 mL of HBSS buffer and incubated at 37°C for 15 min. They were then transferred in culture well containing 1.5 mL of HBSS buffer and incubated at 37°C for 20 min. Concentrations of LY in the wells were determined using a fluorescent spectrophotometer (Berthold, TriStar^2^ LB 942). The emission at 535 nm was measured with an excitation light at 485 nm. The endothelial permeability coefficient of LY was calculated in centimeters/min (cm/min), as previously described [30].

### Virus infections

hCMEC/D3 cells (10^5^) were seeded on coverslips in 24-well tissue culture plates in EndoGro medium supplemented with 5% FBS. After 5 days, cell medium was replaced with 1 mL of EndoGro medium supplemented with 2% FBS. SK-N-SH cells (10^5^) were seeded on coverslips in 24-well tissue culture plates in DMEM supplemented with 2% FBS. Aliquots of virus were diluted in 200μL of medium and added to the cells. Plates were incubated for 1h at 37°C. Unabsorbed virus was removed and 1mL of EndoGro or DMEM supplemented with 2% FBS was added to the cells, followed by incubation at 37°C until collection.

### Immunofluorescence analysis (IFA)

All the following steps were performed at room temperature. Cells were fixed with 4% paraformaldehyde for 20 min followed by permeabilization with 0.1% Triton X-100 for 5 min. After permeabilization, the cells were washed with PBS and incubated for 5 min with PBS containing 1% BSA. The cells were then washed with PBS and incubated for 1h with anti-JEV NS5 antibody diluted at 1:200 in PBS, followed by incubation with Alexa Fluor 488-conjugated anti-mouse IgG antibody diluted at 1:500 in PBS. The coverslips were mounted with ProLong gold antifade reagent with DAPI (Life Technologies; catalog no. P36931). The slides were examined using a fluorescence microscope (EVOS FL Cell Imaging System).

### Gene expression studies

hCMEC/D3 cells (5.10^4^/well) were seeded on 12-well Transwell® insert filters in EndoGro medium supplemented with 5% FBS for 5 days. SK-N-SH cells (2.10^5^/well) were seeded in 12-well tissue culture plates in EndoGro supplemented with 2% FBS. Transwell^®^ containing hCMEC/D3 cells were then transferred in these culture plates and medium was replaced by EndoGro medium supplemented with 2% FBS. Cells were incubated at 37°C. At 24h post-contact, total RNA of hCMEC/D3 cells were extracted using NucleoSpin RNA kit (Macherey-Nagel; catalog no. 740955.50) following the manufacturer’s instructions. Two hundred ng of total RNA were used to produce cDNA using the SuperScript II Reverse Transcriptase (Thermo Fisher; catalog no. 18064014) according to the manufacturer’s instructions. Quantitative PCR were performed on 2μL of cDNA using SYBR Green PCR Master Mix (Thermo Fisher; catalog no. 4309155) according to the manufacturer’s instructions. The CFX96 real-time PCR system (Bio-Rad) was used to measure SYBR green fluorescence with the following program: an initial PCR activation at 95°C (10 min), 40 cycles of denaturation at 95°C (15s) and annealing-extension at 60°C (1 min). Results were analyzed using the CFX Manager Software (Bio-Rad) gene expression analysis tool. GAPDH was used as the reference gene. Primers used in gene expression studies are listed in Table 1.

**Table 1:** Primers used for quantification of receptor, transporter and cytokine encoding genes.

Gene	Forward primer	Reverse primer	Reference
TFRC	5’-ATG CTG ACA ATA ACA CAA-3’	5’-CCA AGT AGC CAA TCA TAA-3’	[31]
AGER	5’-CTC GAA TGG AAA CTG AAC AC-3’	5’-CTG GTA GTT AGA CTT GGT CTC-3’	[31]
LRP1	5’-GCA TCC TGA TCG AGC ACC TG-3’	5’-GCC AAT GAG GTA GCT GGT GG-3’	[31]
INSR	5’-TGT TCA TCC TCT GAT TCT CTG-3’	5’-GCT TAG ATG TTC CCA AAG TC-3’	[32]
LEPR	5’-GGA AAT CAC ACG AAA TTC AC-3’	5’-GCA CGA TAT TTA CTT TGC TC-3’	[32]
BCAM	5’-GCT TTC CTT ACC TCT AAA CAG-3’	5’-GAA GGT GAT AGA ACT GAG CG-3’	[32]
SLC6A8	5’-TGA GAG AAT GAG ATT TCT GCT TGT-3’	5’-TAG GGC TCA CAG GGA TGG-3’	[31]
SLC3A2	5’-TTG GCT CCA AGG AAG ATT-3’	5’-GAG TAA GGT CCA GAA TGA CA-3’	[31]
SLC2A1	5’-GAG ACA CTT GCC TTC TTC-3’	5’-GCT TTG TAG TTC ATA GTT CG-3’	[31]
SLC7A5	5’-TTG ACA CCA CTA AGA TGA T-3’	5’-GTA GCA ATG AGG TTC CAA-3’	[31]
SLC7A1	5’-CCT CCT GAG ACA TCT TTG-3’	5’-CTG GAA TAT GAC GGG AAG-3’	[31]
SLC16A1	5’-ACA CAA AGC CAA TAA GAC-3’	5’-ACA GAA TCC AAC ATA GGT A-3’	[31]
ABCB1	5’-GCC TGG CAG CTG GAA GAC AAA TAC ACA AAA TT-3’	5’-CAG ACA GCA GCT GAC AGT CCA AGA ACA GGA CT-3’	[31]
ABCG2	5’-TGG CTG TCA TGG CTT CAG TA-3’	5’-GCC ACG TGA TTC TTC CAC AA-3’	[31]
ABCC1	5’-ACC AAG ACG TAT CAG GTG GCC-3’	5’-CTG TCT GGG CAT CCA GGA T-3’	[31]
ABCC2	5’-CCA ATC TAC TCT CAC TTC AGC GAG A-3’	5’-AGA TTC CAG CTC AGG TCG GTA CC-3’	[31]
ABCC4	5’-AAG TGA ACA ACC TCC AGT TCC A-3’	5’-CCG GAG CTT TCA GAA TTG AC-3’	[31]
ABCC5	5’-AGT GGC ACT GTC AGA TCA AAT T-3’	5’-TTG TTC TCT GCA GCA GCA AAC-3’	[31]
STRA6	5’-TTT GGA ATC GTG CTC TCC G-3’	5’-AAG GTG AGT AAG CAG GAC AAG-3’	[32]
SLC38A5	5’-TGT CAG TGT TCA ACC TCA G-3’	5’-GTG GAT GGA GTA GGA CGA-3’	[32]
SLC1A1	5’-GTT ATT CTA GGT ATT GTG CTG G-3’	5’-CTG ATG AGA TCT AAC ATG GC-3’	[32]
PLVAP	5’-CAA TGC AGA GAT CAA TTC AAG G-3’	5’-ACG CTT TCC TTA TCC TTA GTG-3’	[32]
CXCL8	5’-TCT TGG CAG CCT TCC TGA TT-3’	5’-TTA GCA CTC CTT GGC AAA ACT G-3’	[33]
CXCL10	5’-TGG CAT TCA AGG AGT ACC TCT C-3’	5’-CTT GAT GGC CTT CGA TTC TG-3’	[34]
GAPDH	5’-AGC CAC ATC GCT CAG ACA CC-3’	5’-GTA CTC AGC GCC AGC ATC G-3’	[31]

### Quantification of JEV RNA copy number

Total RNA from JEV BBB-crossing samples was extracted using NucleoSpin®RNA kit (Macherey-Nagel; 740955.50) according to the manufacturer’s instructions. The number of JEV RNA copies present in BBB-crossing samples was determined by RT-qPCR using TaqMan® Fast Virus 1-Step Master Mix kit (Applied Biosystems®, 4444432) according to the manufacturer’s instructions. The forward and reverse primers (Sigma-Aldrich®) were 5’GAAGATGTCAACCTAGGGAGC3’ and 5’TGGCGAATTCTTCTTTAAGC3’ respectively, while [6FAM]AAGAGCCGTGGGAAAGGGAGA[BHQ1] was the probe for the assay. JEV RNA copies were calculated from a standard curve generated by amplifying known amounts of *in vitro*-transcribed RP9 NS5 gene region cloned and under SP6 promotor control. The *in vitro* transcription was performed using mMESSAGE mMACHINE™ SP6 kit (Invitrogen, Thermo Fisher Scientific, AM1340) following the manufacturer’s instructions.

### Statistical analysis

Unpaired two-tailed *t* test, Mann-Whitney test and ANOVA test corrected with Tukey method for multiple comparisons were used to compare experimental data. GraphPad Prism 7 was used for these statistical analyses. The significance level for our data was set to 5% or less (P ≤0.05).

## Results

### hCMEC/D3 cell monolayers grown on permeable inserts form a BBB whose properties are not affected by SK-N-SH cell presence

**Fig 1:**
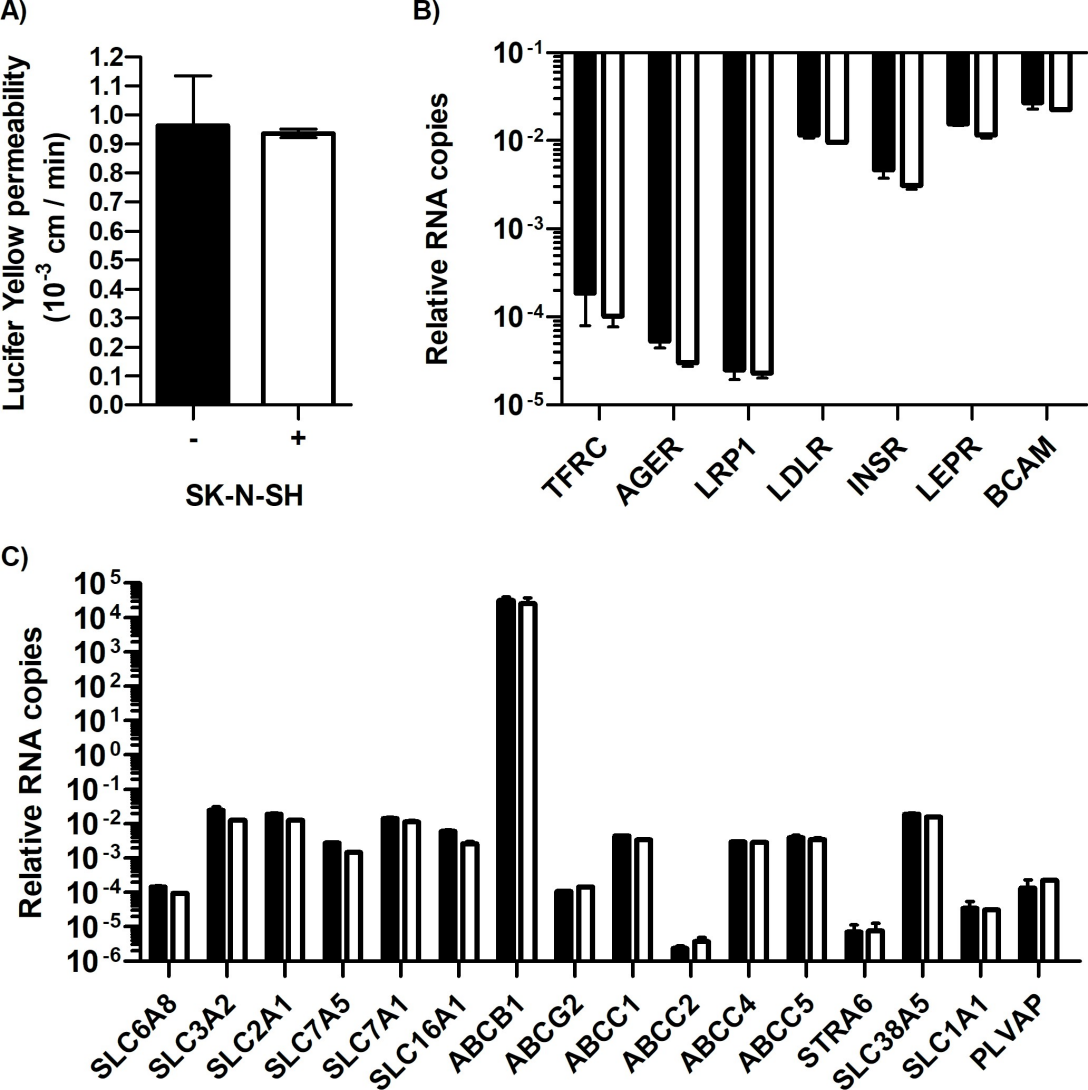
SK-N-SH cells do not affect hCMEC/D3 BBB properties. hCMEC/D3 were cultivated on Transwell^®^ inserts. Five days after seeding, SK-N-SH (SK) cells were cultivated or not in wells under the Transwell^®^ inserts (white and black bars respectively). **A)** Twenty-four hours after adding the SK-N-SH cells (+) or not (-), BBB permeability to LY was measured. **B)** and **C)** hCMEC/D3 BBB total RNA was extracted and expression of receptor (B) and transporter (C) genes typical of the BBB- encoding genes were quantified by RT followed by qPCR as described in Material and Methods. Graphs show the results from two independent experiments performed in duplicates.

A basic *in vitro* model to study JEV neuroinvasion should consist of two main components: 1) a cell monolayer mimicking the BBB, and 2) a brain tissue-derived cell line permissive to JEV. Based on our previous work [26], we chose to use hCMEC/D3 human endothelial cells monolayers cultivated on permeable inserts and place these inserts in wells in which human neuroblastoma SK-N-SH cells were grown, in order to partly mimick the brain parenchyma. Relevant parameters of a functional BBB model, such as permeability and presence of cell transporters and receptors specific of hCMEC/D3 cells were evaluated when the endothelial cells were grown or not above SK-N-SH monolayers (Fig 1). Permeability measurement of hCMEC/D3 monolayers through evaluation of Lucifer Yellow (LY) passage showed no significant difference whether SK-N-SH cells were present or not (Fig 1A, + or—respectively). Moreover, the relative levels of mRNA for genes encoding cell receptors (Fig 1B) and transporters (Fig 1C) characteristic of endothelial barriers were similar in the two conditions. Together, this suggests that the culture of neuroblastoma cells under the inserts on which hCMEC/D3 were grown did not disturb the intrinsic BBB endothelial cell properties and affirms that this *in vitro* BBB model can be used as a tool to study the neuroinvasiveness of JEV.

### JEV SA14-14-2 is less replication efficient than JEV RP9 in SK-N-SH cells

**Fig 2:**
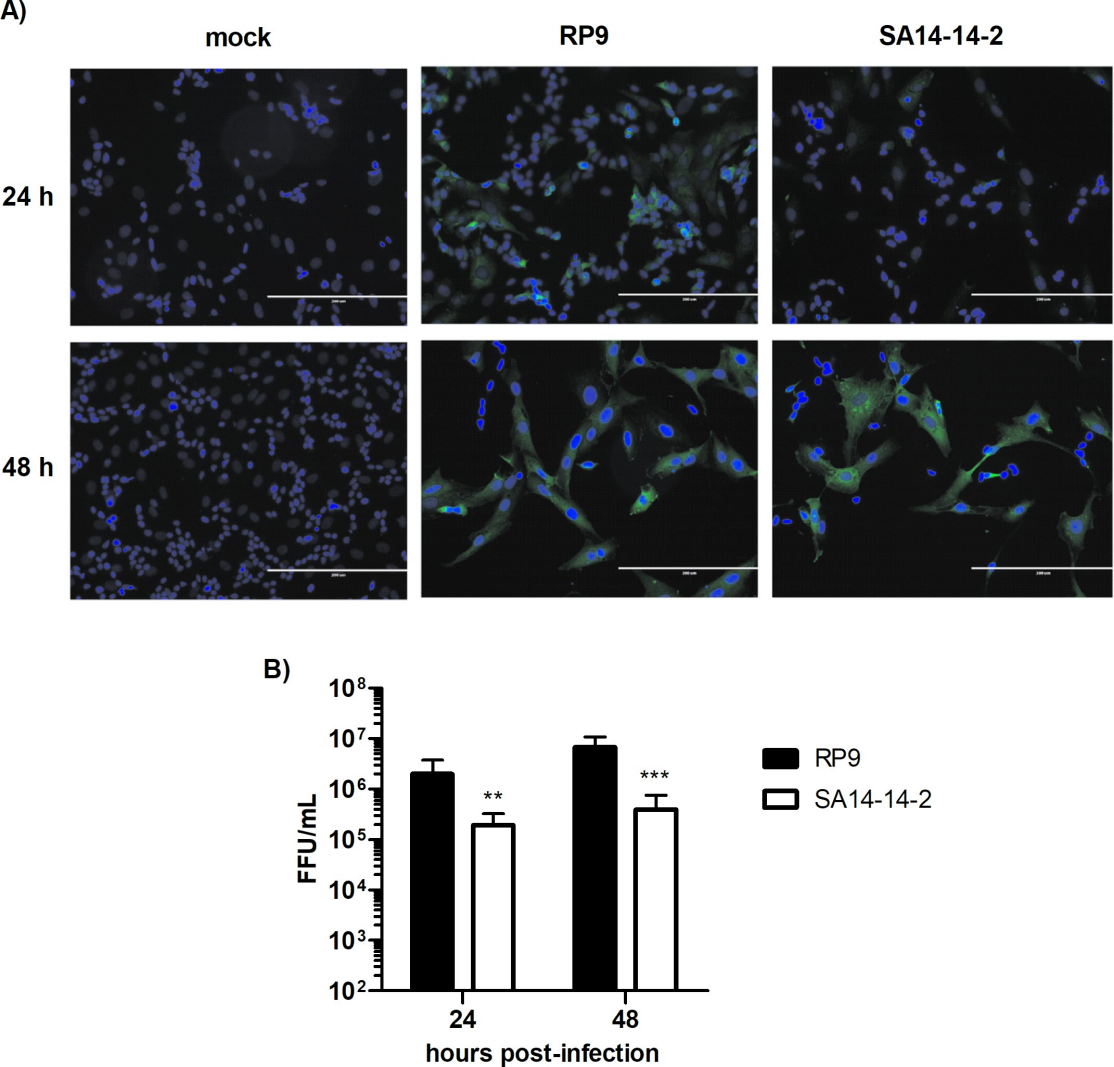
JEV RP9 is more replication efficient than JEV SA14-14-2 in SK-N-SH cells. SK-N-SH cells were infected at MOI 0.1 for 24 or 48h by the indicated JEV strain. **A)** The infected cells were analyzed at the indicated times post-infection by immunofluorescence staining for the presence of the NS5 protein (in green). The images were taken at a x200 magnification (white bars, 100μm), the cell nuclei were stained by DAPI (in blue). **B)** Supernatants of SK-N-SH cells infected by JEV RP9 (black bar) or JEV SA14-14-2 (white bar) were titrated in Vero cells. The arithmetic means ± standard deviation of three independent experiments performed in triplicate is shown. Asterisks indicate a significant difference between RP9 and SA14-14-2 in each one of the times post-infection evaluated (**, P = 0.0023, ***, P < 0.001).

Independent reports have found that neuroblastoma-derived SK-N-SH cells are susceptible to both the virulent JEV RP9 strain and the SA14-14-2 attenuated strain [28, 35]. To directly compare replication of these two JEV strains in SK-N-SH cells, however, we evaluated replication of each JEV strain in these cells at 24 and 48 hpi (Fig 2). As demonstrated by the detection of a viral antigen (NS5 protein) through immunofluorescence assays, both JEV strains infected the SK-N-SH cells but the viral progeny of JEV SA14-14-2 vaccine strain produced in SK-N-SH cells at 24 and 48 hpi was significantly lower than that of JEV RP9 (1.7 and 1.2 log_10_ less at 24 and 48 hpi respectively, Fig 2B), suggesting that JEV SA14-14-2 is less neurovirulent than JEV RP9 in human cell cultures.

### Neither JEV RP9 nor JEV SA14-14-2 infects hCMEC/D3 cells after they form a BBB

**Fig 3:**
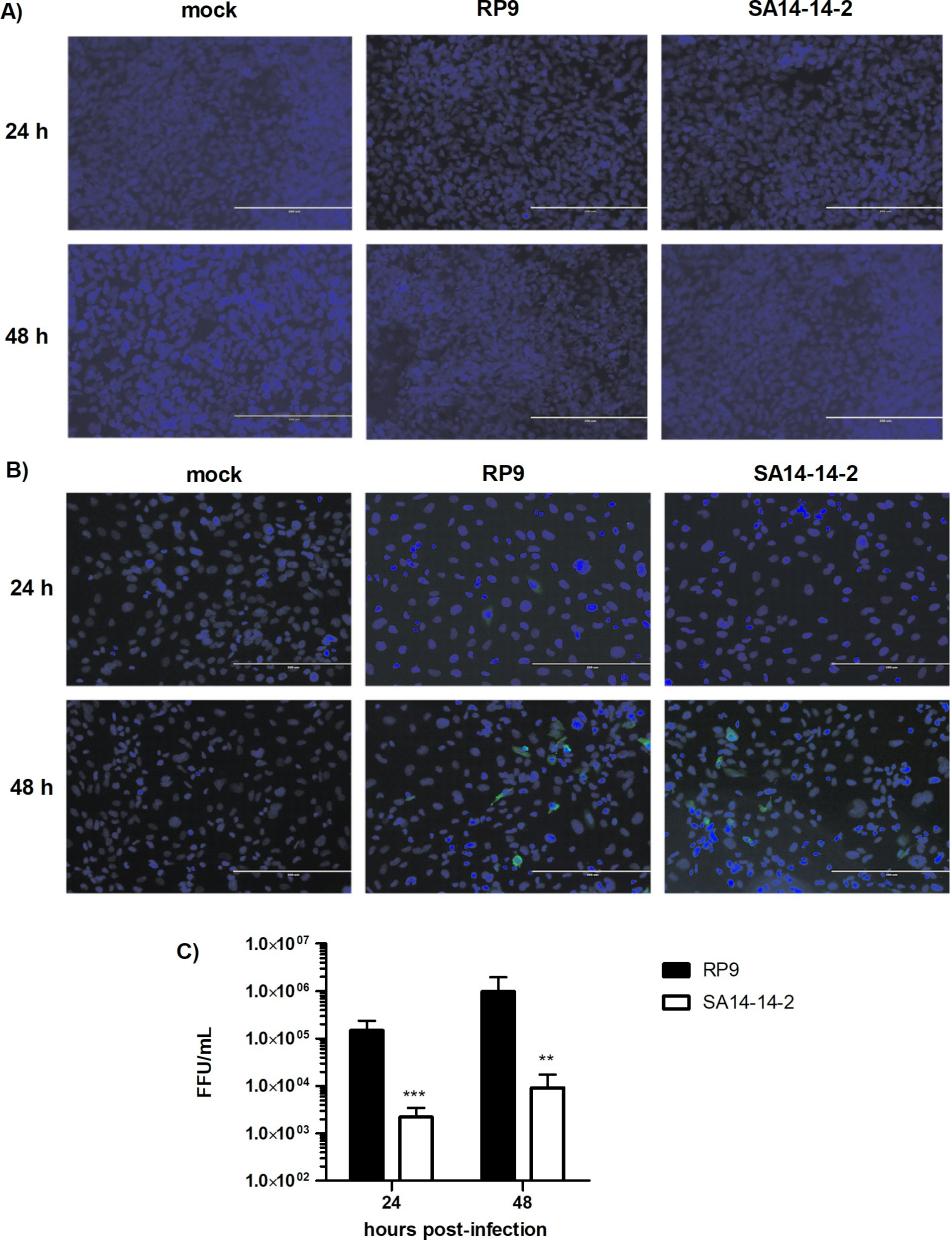
Infection of hCMEC/D3 cells by JEV strains. hCMEC/D3 were cultured on coverslips for either 6 days (**A**) or 1 day (**B**), so that they form or do not form a BBB respectively. Cells were then inoculated with the indicated JEV strain at MOI = 0.1 and analyzed at 24 and 48 hpi by immunofluorescence staining for the presence of the NS5 protein (in green). The images were taken at a x200 magnification, cells nuclei are stained by DAPI (in blue). **C)** Supernatants from non-forming BBB hCMEC/D3 cells infected by JEV RP9 (black bar) or JEV SA14-14-2 (white bar) were collected at 24 and 48 hours post-infection and their viral titer was determined as described in Material and Methods. The arithmetic means ± standard deviation of three independent experiments performed in triplicate is shown. Asterisks indicate a significant difference between RP9 and SA14-14-2 for each time post-infection evaluated (**, P = 0.0056, ***, P < 0.001).

In order to examine the susceptibility of our hCMEC/D3 BBB model to JEV infection, the cells were grown 6 days on coverslips to allow the BBB to form, and then inoculated with either the RP9 or SA14-14-2 JEV strain (Fig 3). As evidence of infection we assessed the expression of NS5 viral protein by immunofluorescence microscopy (see Material and Methods) and none was observed at either 24 or 48 hpi (Fig 3A). On the other hand, hCMEC/D3 cells could be infected by either JEV strains when they were inoculated after only one day of culture (ie not forming of a BBB), as detected through the same immunofluorescence approach (Fig 3B). Moreover, in this condition, both JEV strains produced infectious viral progeny in hCMEC/D3, although the RP9 viral titer was significant higher by around 2 log than that observed for SA14-14-2 (Fig 3C). These results suggest that hCMEC/D3 cells are not susceptible to JEV infection when they already have formed a barrier, but they are JEV permissive before tight junction formation.

### Neither JEV RP9 nor JEV SA14-14-2 disrupt the BBB when added for 6h

**Fig 4:**
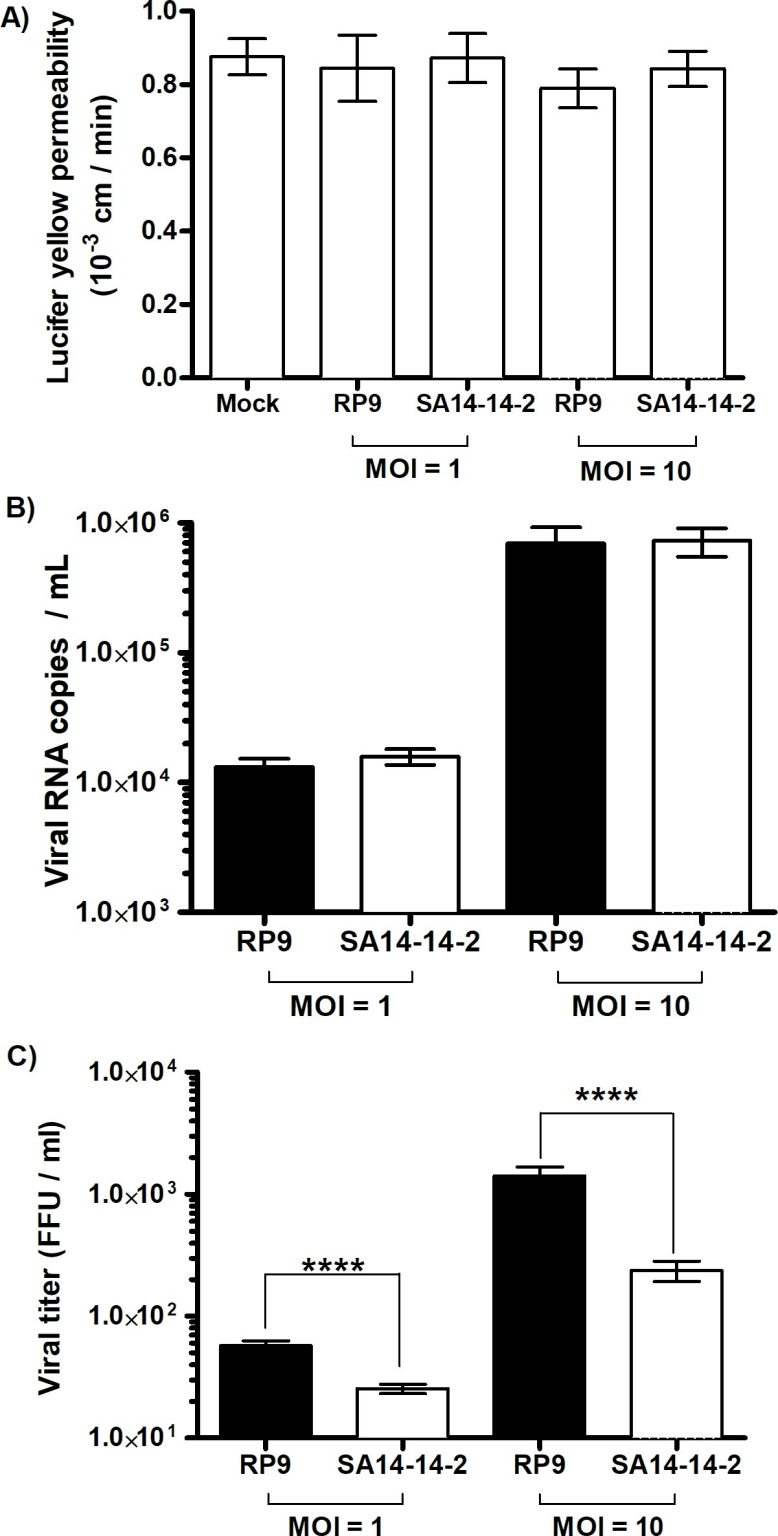
JEV RP9 and JEV SA14-14-2 may cross the *in vitro* BBB model without disrupting it. A) hCMEC/D3 cells were cultivated on Transwell^®^ inserts. Five days after seeding, SK-N-SH cells were added to the wells under the Transwell^®^ insert. JEV RP9 or SA-14-14-2 was added to the BBB as indicated 24h later, either at MOI = 1 or = 10. hCMEC/D3 cell permeability to Lucifer Yellow was assayed 6 h post-addition as described in Material and Methods. B) *In vitro* BBBs were generated as indicated above and either JEV strains were added at MOI = 1 or = 10. After 6 h, total RNA was extracted from media under the inserts and the number of JEV RNA copies was determined by RT-qPCR as described in Material and Methods. C) Samples were collected 6 h post-addition (JEV RP9, black bars or SA14-14-2, white bars) and their viral titer was determined as described in Material and Methods. The arithmetic means ± standard deviation of at least two independent experiments performed in triplicate is shown. Asterisks indicate a significant difference between the RP9 and SA14-14-2 titers for each MOI evaluated in the BBB-crossing experiments (****, P < 0.0001).

It has been suggested that JEV infects brain tissue cells as a consequence of a preceding inflammatory process which in turn leads to disruption of the BBB and viral neuroinvasion [36, 37]. Our knowledge of the very early events of JEV crossing the BBB is, however, still scant. In order to shed some light on these early times of viral exposure, we evaluated the neuroinvasive ability of JEV in our BBB model at early times post viral addition. hCMEC/D3 cells were cultivated on permeable inserts to form a BBB above a SK-N-SH cell monolayer and exposed to either JEV RP9 or SA14-14-2 viruses at MOIs of 1 or 10 (Fig 4). As assayed by Lucifer Yellow permeability, we found the BBB integrity was not significantly compromised by either JEV strain when compared to mock-infected conditions (Fig 4A), suggesting that the BBB model was not disturbed either by the JEV strains or the MOIs used.

### More JEV RP9 infectious particles may cross the *in vitro* BBB model than JEV SA14-14-2

**Fig 5:**
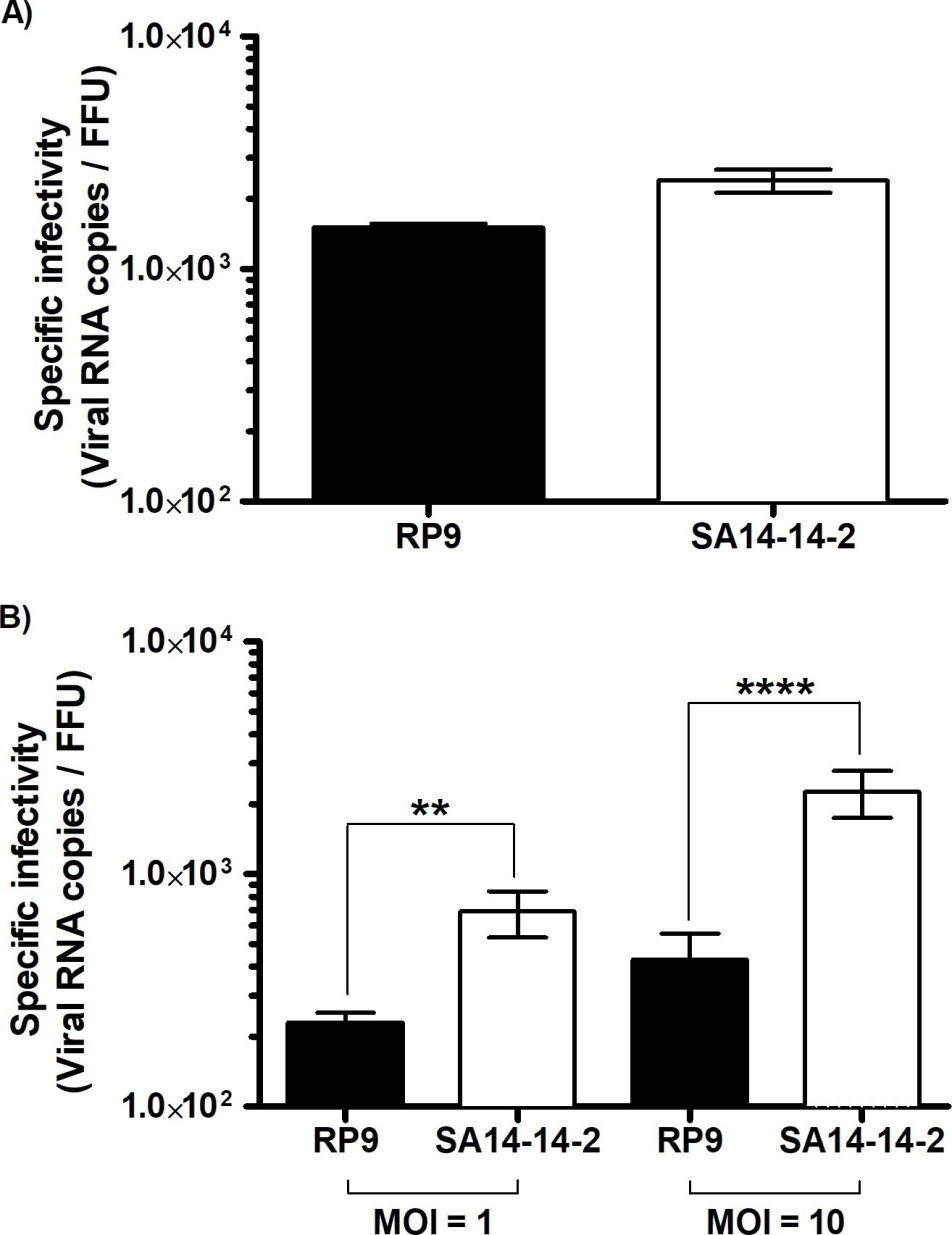
The specific infectivity of JEV RP9 is decreased after BBB-crossing. **A)** Both the number of viral RNA copies and the infectious titers for either JEV RP9 or JEV SA14-14-2 stock used for the JEV BBB-crossing experiments were determined as described in Material and Methods. The specific infectivity of both stocks, calculated by dividing the viral RNA copies number/ml by the FFU/ml of each viral stock, was evaluated after 6h incubation at 37°C. **B)** The specific infectivity of the JEV RP9 and SA14-14-2 BBB-crossing samples was calculated as indicated above using the data from Fig 4B and 4C. The arithmetic means ± standard deviation of at least two independent experiments performed in triplicate is shown. Asterisks indicate a significant difference between the specific infectivity of RP9 and SA14-14-2 for each MOI evaluated in the BBB-crossing experiments (**, P = 0.0022; ****, P < 0.0001).

Since the BBB permeability was not affected by the addition of either virus, we quantitated the viral crossing of each strain by assaying the quantity of viral RNA and infectious particles in the supernatants under the inserts (Fig 4B and 4C). The number of viral RNA copies detected for both viruses was 1.7 log_10_ higher when a MOI of 10 was used in comparison to a MOI of 1 (Fig 4B), suggesting that the higher the JEV viral load, the greater the number of viral particles crossing the BBB. Of note, there was no significant difference in the viral RNA copy number between the JEV strains for each MOI (MOI = 1 or = 10, Fig 4B). However, the infectious titers of the JEV particles that crossed the BBB was notably different between the RP9 and SA14-14-2 strains, as about 3 times more RP9 infectious particles compared to SA14-14-2 where found in the supernatants under the inserts when an MOI of 1 was used, and close to 10 times more for a MOI of 10 (Fig 4C). Calculation of the specific infectivity for JEV RP9 and SA14-14-2 strains as the ratio between the detected JEV RNA copy number per infectious focus-forming unit did not show a significant difference between the 2 viral stocks (Fig 5A). Interestingly, the specific infectivity for the RP9 BBB-crossing samples was significantly lower than that observed for the vaccine strain SA14-14-2 with a 3 to 10 fold decrease for MOI of 1 and 10 respectively (Fig 5B). These results indicate that more JEV RP9 infectious particles may cross our BBB model than SA14-14, and demonstrate that this *in vitro* barrier is capable of discriminating between 2 viruses with different neuroinvasive capabilities.

## Discussion

Lines of research from both *in vivo* and *in vitro* systems have suggested JEV infects brain tissue cells as a consequence of a preceding inflammatory process that in turn may facilitate BBB disruption and viral neuroinvasion [36, 37]. While *in vivo* approaches primarily give insights to systemic viral disease, *in vitro* models tend to allow examination and manipulation of the molecular mechanisms that govern viral pathogenesis. In this regard, previous approaches have generally focused on characterizing JEV neuroinvasive properties at late times of infection, mainly 24 hpi or later [35, 38–40], leaving our knowledge of events at early times of JEV contact with the BBB poor, if not null.

In this study, we have used an *in vitro* human BBB model to compare the ability of two JEV strains (the virulent RP9 strain and the SA14-14-2 vaccine strain) to cross the BBB at early times post-addition. We have shown that both JEV RP9 and SA14-14-2 are able to cross the BBB without disrupting it at 6 hpi. Our finding corroborates *in vivo* studies that have demonstrated that JEV is able to get access to the CNS and establish a primary infection without the preceding need of BBB leakage [36, 37].

Moreover, the fact that both JEV RP9 and SA14-14-2 strains crossed the BBB without infecting BBB endothelial cells, or disrupting the barrier, also suggests that the pathway JEV uses to cross the BBB is either a transcellular one, through the endothelial cells, or paracellular, between the endothelial cells. These observations are consistent with other studies conducted *in vivo* in mice and monkeys [11, 16, 41]. Electron-microscopic studies of brains from JEV-infected suckling mice have suggested that viruses cross the BBB endothelial cells by transcytosis [14]. In spite of these observations, to date there are no published data from biochemical, genetics or functional approaches to support or refute this hypothesis. The combination of these approaches, together with the use of our *in vitro* BBB model and JEV strains with different neuroinvasive capabilities(such as the ones used in this work) would be useful to identify which cellular mechanisms might be "hijacked" by these pathogens to cross the BBB.

Interestingly, although our specific infectivity data suggest that JEV RP9 infectious particles crossed the BBB more efficiently than those of the vaccine strain JEV SA14-14-2, comparison of hCMEC/D3 cell transcriptomes from BBBs that were exposed for 6h to either JEV RP9, SA14-14-2 or no virus showed no significant difference in the levels of gene expression (fold-change threshold of 2, data not shown). This suggests that an immediate or early cellular response is unlikely to be responsible for the differential BBB crossing of JEV RP9 versus JEV SA-14-14-2 particles we observed. Instead, we suspect specific viral factors to be at play, for example, interaction of the viral particle with a strain-specific cellular surface receptor for viral entry. Other considerations to pursue such as full characterization of the viral particles that are able to cross the BBB including, by deep-sequencing of their RNA genomes, and an electron microscopic examination of the endothelial cells forming the BBB after contact with either virus, could help to shed significant light on this intriguing difference.

Interestingly, we found that hCMEC/D3 were permissive to both RP9 and SA14-14-2 strains only when the BBB formation was not completed. BBB formation induces changes in cell conformation, which can then lead to the relocation of cell receptors between BBB cells [42]. Differences in hCMEC/D3 cells permissiveness could be due to differential accessibility of cell receptors when BBB is formed. Based on our data, and considering the current model of JEV neuroinvasion that suggests disruption of the BBB following CNS viral infection [11], endothelial cells from a disrupted barrier might become permissive to JEV because of better accessibility to cell entry receptor(s), and these cells, upon infection, could in turn become a new source of viral production contributing to JEV infection of the CNS.

In conclusion, our study demonstrates that both a virulent and a vaccine strain of JEV are able to cross a BBB model without disruption at early times post viral addition. This BBB formed by human endothelial cells represents a useful discriminant *in vitro* model to characterize viral determinants of JEV neuroinvasiveness as well as a tool to study the molecular mechanisms by which these pathogens cross the BBB.
